# Phage–Antibiotic Therapy as a Promising Strategy to Combat Multidrug-Resistant Infections and to Enhance Antimicrobial Efficiency

**DOI:** 10.3390/antibiotics11050570

**Published:** 2022-04-25

**Authors:** Chengxi Liu, Qixuan Hong, Rachel Yoon Kyung Chang, Philip Chi Lip Kwok, Hak-Kim Chan

**Affiliations:** Advanced Drug Delivery Group, Faculty of Medicine and Health, School of Pharmacy, The University of Sydney, Sydney, NSW 2006, Australia; cliu8026@uni.sydney.edu.au (C.L.); qhon6502@uni.sydney.edu.au (Q.H.); yoon.chang@sydney.edu.au (R.Y.K.C.); philip.kwok@sydney.edu.au (P.C.L.K.)

**Keywords:** antibiotic, bacteriophage, phage, PAS, MDR, phage–antibiotic therapy

## Abstract

Infections caused by multidrug-resistant (MDR) bacteria have highlighted the importance of the development of new antimicrobial agents. While bacteriophages (phages) are widely studied as alternative agents to antibiotics, combined treatments using phages and antibiotics have exhibited Phage–Antibiotic Synergy (PAS), in which antibiotics promote phage replication and extraordinary antimicrobial efficacy with reduced development of bacterial resistance. This review paper on the current progress of phage–antibiotic therapy includes aspects of the mechanisms of PAS and the therapeutic performance of PAS in combating multidrug-resistant bacterial infections. The choice of phages and antibiotics, the administration time and sequence, and the concentrations of the two agents impact the bacterial inhibitory effects to different extents.

## 1. Introduction

Antibiotics are one of the greatest discoveries in modern medicine and have extensively decreased morbidity and mortality caused by different types of bacterial infections, improving the quality of life and life span of patients [1]. However, misuse and overuse of antibiotics have led to multidrug resistance in various bacterial species. *Enterococcus faecium*, *Staphylococcus aureus*, *Klebsiella pneumoniae*, *Acinetobacter baumannii*, *Pseudomonas aeruginosa*, and *Enterobacter* species (the ESKAPE pathogens) have developed resistance to oxazolidinones, lipopeptides, macrolides, fluoroquinolones, tetracyclines, β-lactams, β-lactam–β-lactamase inhibitor combinations, polymyxins, glycopeptides, and carbapenems [2]. In the global priority pathogens list published by the WHO in 2017, *A. baumannii*, *P. aeruginosa*, and *Enterobacteriaceae* are listed as the most critical pathogens that require the development of new antibiotics because they have developed resistance to carbapenem, which is the last-line drug for combating resistant bacteria [2,3]. In Antibiotic Resistance Threats 2019, published by the Centers for Disease Control and Prevention (CDC), 14 MDR bacteria are listed and classified into three categories: urgent, serious, and concerning. Carbapenem-resistant *Acinetobacter*, carbapenem-resistant *Enterobacteriaceae*, and drug-resistant *Neisseria gonorrhoeae* are listed as urgent threats [4]. This antibiotic resistance crisis has compelled scientists to develop new antibiotics or other novel antimicrobial agents. As new antibiotic development requires a huge input of effort and costs, an alternative strategy, using bacteriophages, has regained scientists’ attention.

Bacteriophages (or phages) are viruses that specifically target bacteria [5]. Phage therapy was first explored approximately a century ago [6], but its use was soon stalled due to the discovery of a wide-spectrum antibiotic, penicillin. Phage therapy has been increasingly studied in vitro and in vivo in recent decades due to the rise of antibiotic resistance.

**Figure 1:**
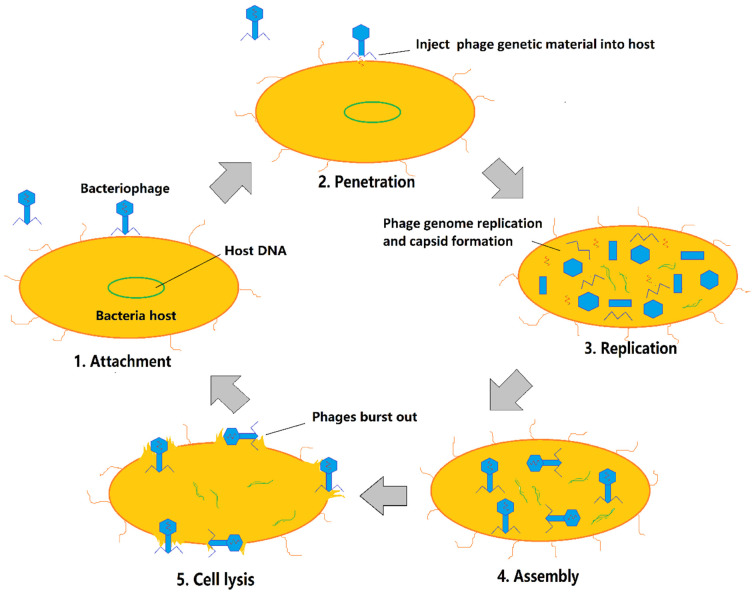
Infection cycle of lytic phage.

Virulent phages (or lytic phages), which are unable to lysogenise their hosts, multiply in bacterial hosts, and lead to bacterial lysis after each infection cycle, are only used for therapeutic investigation against bacterial infections. Either wild-type lytic phages [7,8] or engineered lytic phages [9] have been used clinically. Engineered phages are necessary, especially when naturally occurring lytic phages are not available. The lytic infection cycle (Figure 1) begins with phage adsorption to the host cell by binding to specific receptors, including lipopolysaccharides (LPS), teichoic acids, membrane proteins, and capsules [10,11]. The translocation of phage genetic material then happens; phage genetic material is injected into the host, and the replication machinery is hijacked to produce phage progeny. The activation of the phage-encoded protein drives the bacteriolysis, and the progeny escapes the bacterial cell to re-initiate the cycle on other hosts [12].

Antibiotics are chemical compounds that have a specific mechanism for disrupting either the cell wall or the intracellular pathways. For example, carbapenem inhibits cell wall formation, and ciprofloxacin inhibits DNA replication. Because the mechanisms for phages to inhibit bacteria are different from those of antibiotics, phages can treat MDR bacterial infections. However, bacteria can still evolve an antiphage system in a number of ways: (1) mutating bacterial cell surface receptors, preventing the entry of phage DNA [13,14]; (2) editing foreign (phage) DNA with clustered, regularly interspaced short palindromic repeats (CRISPR) and CRISPR-associated genes, forming a restriction-modification system in which enzymes in the host bacteria recognise and cleave the foreign phage DNA to stop the invasion [15,16]; aborting the infection by causing bacterial hosts to commit “suicide” [17]; and interfering with the assembly of phages [18]. As a remedy for the potential development of resistance to single antibacterial agents, scientists have extended mono-phage therapy to include a combination therapy using phages and antibiotics.

Phage–antibiotic therapy has been successfully shown to reduce the emergence of phage-resistant and antibiotic-resistant strains. An often-cited example is the fact that the degeneration of cell surface receptors caused by phages restores antibiotic sensitivity, as those receptors are responsible for the efflux of antibiotics [19]. Several in vitro studies corroborate the re-sensitisation to antibiotics when combined with phages to reduce bacterial concentration [20,21].

As a promising therapeutic strategy for bacterial infection, the combination therapy using phages and antibiotics has now been studied extensively, not only because of its improved performance in reducing phage and antibiotic resistance but also for the synergistic antibacterial effects achieved by phage replication enhancement in the presence of antibiotics (the so-called Phage–Antibiotic Synergy, PAS) [22,23].

This review focuses on the PAS response and its mechanisms; the development of resistance in bacteria in response to phage–antibiotic combined treatment; and the application of phage–antibiotic treatment in vitro, in vivo, and in clinical case studies.

## 2. Phage–Antibiotic Synergy (PAS)

### 2.1. Antibiotic-Enhanced Phage Production

PAS has been observed between phages and antibiotics. This has raised interest in the therapeutic potential of PAS to improve bacterial killing [24,25].

The term PAS initially described the phenomenon of the improved antimicrobial effect caused by stimulated phage replication in the presence of sublethal concentrations of antibiotics. Nowadays, the term PAS is used in circumstances where synergistic antimicrobial effects occur. In this review, we use the term PAS to describe the phenomenon of increased phage activity in the presence of sublethal concentrations of antibiotics. The terms synergistic effects or synergism are used herein to describe the significant improvement in bacterial growth inhibition with phage–antibiotic co-treatment.

PAS was first studied by Comeau et al. [23], where significant plaque size enlargement was observed on coliphage ФMFP against *Escherichia coli* clinical isolate MFP; on T4-like phages against standard laboratory *E. coli* strains; on T3 and T7 phages against laboratory *E. coli* strain AS19; and on T4-type *Yersinia* phage PST against *Yersinia pseudotuberculosis* in the presence of sublethal β-lactams such as aztreonam, cefotaxime, ticarcillin, piperacillin, ampicillin, and quinolones such as nalidixic acid. The plaque size enlargement implies enhanced phage production during plaque formation, resulting in increased bacteria growth inhibition and/or lysis. Other than *E. coli* phage and *Y. pseudotuberculosis* phage [23], plaque size enlargement was also seen in phages specific to *P. aeruginosa* [26,27], *S. aureus* [27,28,29], *Bacillus cereus* [27], *Enterococcus faecalis* [27], and *Burkholderia cepacian* [30].

**Figure 2:**
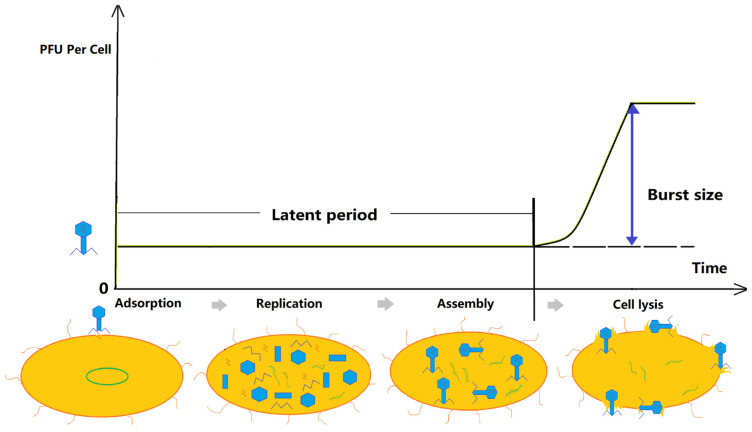
One-step multiplication curve of phage.

Plaque size can be affected by multiple factors, including the intrinsic traits of the phage and the infected host, as well as extrinsic factors such as incubation time, temperature, agar density, and bacterial density [31]. As these parameters were all fixed in the study, phage plaque enlargement in the presence of sub-lethal concentrations of antibiotics was driven by changes in the intrinsic traits of the phage or bacterial host, which in turn would alter phage–bacteria interactions. During the lytic cycle of phages (Figure 1), the properties of the phage (Figure 2), including adsorption rate (Section 2.2.1), burst size (Section 2.2.3), and latent period (Section 2.2.2), play a critical role in determining the plaque size. Plaque size is proportional to the adsorption rate and burst size and inversely proportional to the latent period [32,33,34,35,36]. In this section, we discuss the effects of antibiotics on bacteria and the subsequential effects on adsorption rate, latent period, and burst size.

### 2.2. PAS Induced by Bacterial Filamentation

The PAS response in lytic phages is considered directly related to bacterial filamentation, which increases the bacterial size and cellular surface area [26,27,37,38]. The plaque diameter of phage E79 increased 1.6-fold in the presence of 1.06 μg/mL aztreonam lysine and was accompanied by the elongation of the host bacteria *P. aeruginosa* PA01 from an average of 1.15 ± 0.18 μm to 1.8 ± 0.18 μm (i.e., ~1.6-fold) [26].

In another PAS study, 11 phages in combination with 8 antibiotics (ampicillin, cefotaxime, kanamycin, tetracycline, ciprofloxacin, mitomycin C, trimethoprim, and sulfamethoxazole) were tested against their hosts: phage SA11 against *S. aureus* ATCC 12201; PBEF7 and PBEF9 against *E. faecalis* KCTC 2011; PBBC03 against *B. cereus* KCTC 1012; T4 against *E. coli* K-12 strain ATCC 700926; PBEC22, PBEC24, and PBEC82 against *E. coli* Crooks strain ATCC 8739; and PA26, PA22, and PA25 against *P. aeruginosa* ATCC 13388. Of the 88 tested phage and antibiotic combinations, 56 exhibited an increase in plaque size along with antibiotic-induced bacterial filamentation in rods or bacterial swelling in cocci [27]. The prevalence of the PAS response in filamented bacterial hosts supported a strong correlation between PAS and bacterial filamentation. The only exception was tetracycline with *P. aeruginosa* phage PA22, in which PAS did not occur (i.e., no plaque size increase), yet the bacteria filamented. However, the reason remains unclear.

Most of the antibiotic-induced filamentation can be explained by two mechanisms: the β-lactam-induced and SOS-response-mediated mechanisms. β-lactams alter the morphology of bacteria by binding to penicillin-binding proteins (PBPs) on the bacterial surface. PBPs are key peptidoglycan-synthesizing enzymes that play an integral role in constructing the bacterial cell wall. For example, in *E. coli* strains, PBP1s are involved in bacterial elongation, PBP2s are responsible for cell elongation and rod shape maintenance, and PBP3s are responsible for septal wall formation during cell division. Blocking PBP1s leads to direct cell lysis, blocking PBP2s leads to ovoid bacterial cells, and blocking PBP3s leads to bacterial filamentation [39].

β-lactams vary in their affinity for different PBPs, which induce different alterations in bacterial shapes to different extents or result in direct cell lysis [39]. β-lactams such as ceftazidime and cefuroxime have higher affinity for PBP3s, which are required for septum formation between daughter bacterial cells during division in *E.coli* and *P. aeruginosa* cells [40,41]. In the presence of ceftazidime or cefuroxime, PBP3s are blocked, and bacterial cells remain filamented/elongated instead of dividing [40,41,42]. *E. coli* and *P. aeruginosa* can form filaments in 3–4 h in the presence of sublethal concentrations of cefuroxime and ceftazidime (0.008 × MIC~1 × MIC) [40,41]. On the contrary, in *E. coli* and *P. aeruginosa* cells, β-lactams such as imipenem and mecillinam have higher affinity for PBP2s and are reported to cause ovoid cells; ampicillin has similar affinity for PBP2s and PBP3s and is reported to cause localised swelling [43] as well as filamentation [27]. Other than the filamentation in rods and swelling in cocci [27], PAS response is not reported to be associated with other forms of morphological change.

Consequently, the choice of a specific β-lactam antibiotic affects the degree of filamentation, which in turn may affect the adsorption rate of phages by increasing phage targeting receptors on the bacterial cell wall (for details, see Section 2.2.1).

Besides β-lactam-induced filamentation, the SOS response can also induce bacterial filamentation when bacterial DNA is damaged or DNA synthesis is inhibited. The bacterial SOS response inhibits cell division to avoid damaged DNA being transmitted to the progeny. Similar to β-lactam-induced filamentation, the bacterial septum formation is suppressed until the bacterial DNA is repaired. Bacterial size keeps growing while division is inhibited, which leads to bacterial filamentation. Examples of SOS-response-mediated filamentation include DNA synthesis inhibitors such as fluoroquinolones [44], mitomycin C [45]), and other agents that disrupt DNA by other routes, such as folic acid synthesis inhibitors (e.g., trimethoprim [46]).

It is generally believed that the observed PAS response with quinolones is due to filamentation induced by the SOS response. However, exceptions were reported by Kim et al. [27]. *P. aeruginosa* did not exhibit filamentation even though *recA* expression (gene *recA* was monitored to detect the SOS response; its resulting protein product RecA is involved in DNA repair when the SOS response is triggered) was detected in the presence of a sublethal dose of ciprofloxacin, whereas other tested bacteria (*S. aureus*, *E. faecalis*, *B. cereus*, *E. coli* K-12, and *E. coli* Crooks) exhibited both *recA* expression and filamentation [27]. Moreover, *recA* deletion *E. coli* mutant strain showed filamentation and PAS in the presence of ciprofloxacin even with no *recA* expression. These results suggest that the SOS response may not necessarily be responsible for bacterial filamentation induced by DNA synthesis inhibitors or DNA disrupting agents; filamentation could also be triggered through other pathways.

Moreover, although SOS-response-mediated filamentation has been reported in studies with mono-treatment of trimethoprim and sulfamethoxazole [43], conflicting results by Kim et al. [27] reported no filamentation observed in sulfamethoxazole against *S. aureus*, *E. faecalis*, *B. cereus*, *E. coli* K-12, *E. coli* Crooks, or *P. aeruginosa*, or in trimethoprim against *E. coli* K-12. One possible reason is that SOS-response-mediated filamentation did not happen at the tested antibiotic concentration or within the exposure period (not specified in the article) as filamentation is affected not only by the antibiotic class but also by the antibiotic concentration and duration of exposure [43]. As the *recA* expression was only tested in ciprofloxacin, it is unknown whether the SOS response is triggered by trimethoprim and sulfamethoxazole.

The SOS response was also found to improve temperate phage activity because it triggers temperate phage assembly and release due to the cleavage of phage repressors by RecA [47,48]. We have not widely discussed this in this section because the main subjects for PAS studies are lytic phages, which do not lysogenise their hosts (see Section 2.3 for information about the SOS response activating temperate phage release).

Although PAS and filamentation have rarely been observed with sublethal concentrations of antibiotics other than β-lactam and DNA-disrupting antibiotics [23,27], filamentation at lethal concentrations was reported for antibiotics with mechanisms of protein synthesis inhibition (e.g., chloramphenicol against *Bacterium coli* [49], aminoglycosides against *P. aeruginosa* [50]) or RNA synthesis inhibition (e.g., bicyclomycin against *E. coli* [51]). Although the mechanism is yet to be elucidated, bacterial filamentation induced by protein synthesis inhibitor or RNA synthesis inhibitor tends to produce shorter cell lengths than those induced by β-lactam and fluoroquinolone. These differences in the changes to the surface area of the bacterial cell may in part explain why β-lactams and fluoroquinolones are more frequently observed than other antibiotics in PAS [23,24,27].

**Figure 3:**
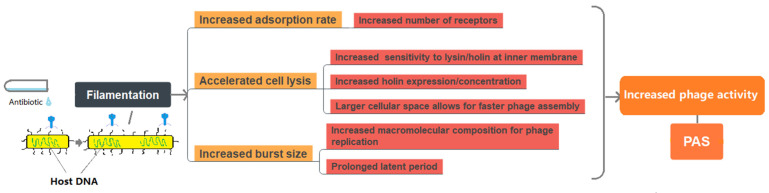
Overview of the factors that may contribute to PAS responses.

In addition to structural changes in bacterial cells (i.e., elongation), some alterations in the phage lytic cycle were found in the presence of filamentation and the PAS response. To date, three main alterations in the phage lytic cycle have been reported to induce the PAS response (Figure 3). It is usually the interplay between more than one alteration that affects phage multiplication.

PAS may be due to bacterial filamentation and resultant changes in the phage lytic cycle (Section 2.2.1, Section 2.2.2 and Section 2.2.3), but filamentation alone does not always result in PAS.

Filamentation occurred either when PBPs were inhibited by β-lactam antibiotics (β-lactam-induced) or as a survival mechanism when DNA was damaged by antibiotics (SOS-response-mediated). When bacteria remain filamented after antibiotic treatment, genome replication and expression continue without separation into daughter cells. Once antibiotics are depleted or the DNA is repaired, bacterial division and replication resume. If, before the antibiotics are depleted, the phages lyse all bacteria cells with only limited numbers of the lytic cycle, no significant phage replication is observed. This does not provide an advantage to phage multiplication, hence no PAS. However, due to early bacterial clearance, phage–antibiotic co-therapy is still beneficial because the phages promote bacterial lysis.

Since phage–antibiotic therapies are mainly used for combating MDR infection, it is expected that MDR bacteria cannot be fully killed/inhibited by antibiotics alone and that they continue to grow in the presence of antibiotics; consequently, the phages undergo replication. In addition, if the antibiotic dose is too low for a subinhibitory effect against the bacteria, or the bacteria are highly resistant to the antibiotics and filamentation cannot be induced, PAS may not occur. Thus, PAS may hinge on a balance between the bacterial replication rate in the presence of a sublethal concentration of antibiotics and the phage replication rate to lyse the host. As a result, the choice of the type and dose of antibiotics for a given phage is a critical determinant of the PAS response.

#### 2.2.1. Improved Phage Adsorption Rate

Phage adsorption to bacteria, as the first stage of the lytic cycle, is one of the critical steps for phage multiplication. Phage adsorption includes reversible and irreversible binding. Phage adsorption rate refers to irreversible binding.

An improvement in phage adsorption efficiency was found in *E. coli*, *S. aureus*, and *P. aeruginosa* in the presence of sublethal concentrations of antibiotics. By pre-treating *E. coli* B/r H266 with a low concentration of penicillin, the adsorption rate of T4 phages was significantly increased (4.68 × 10^7^ phage/mL/min) compared to the control (3.36 × 10^7^ phage/mL/min) [52].

Kaur et al. [29] measured the time needed for 50% (T_50_), 75% (T_75_), and 100% (T_100_) of phage MR-5 to be adsorbed to *S. aureus* ATCC 43300 pre-treated with antibiotics for 90 min (1 µg/mL linezolid, 0.25 µg/mL tetracycline, 4 µg/mL clarithromycin, and 4 µg/mL telithromycin) and a control bacteria culture. Pre-treatment with linezolid and tetracycline significantly decreased T_50_ (from 3.0 ± 0.52 min to 1.7 ± 0.2 and 1.4 ± 0.31 min, respectively), T_75_ (from 7.0 ± 0.64 min to 3.7 ± 0.21 and 3.8 ±0.25 min, respectively), and T_100_ (from 21.3 ± 1.15 min to 14.6 ± 1.12, 15.3 ± 1.15 min, respectively), while clarithromycin and telithromycin significantly decreased T_50_ (from 7.0 ± 0.64 min to 2.0 ± 0.50 and 2.0 ± 0.28 min, respectively) and T_75_ (from 7.0 ± 0.64 min to 3.5 ± 0.25 and 4.0 ± 0.36 min, respectively).

Another assessment of the filamentation and improved attachment of phage E79 to *P. aeruginosa* PA01 in the presence of 1.06 µg/mL aztreonam lysine was conducted using transmission electron microscopy (TEM). The cell significantly increased in length (control cells: 1.15 ± 0.18 μm; aztreonam lysine-treated: 1.8 ± 0.18 μm) and width (control cells: 0.51 ± 0.05 μm; aztreonam lysine-treated: 0.55 ± 0.03 μm). Meanwhile, approximately a two-fold increase in phage attachment was detected in the presence of aztreonam lysine: an average of 2 ± 1 phage E79 adsorbed to each *P. aeruginosa* PA01 cell in the control, while 5 ± 2 phages per cell were detected in an aztreonam lysine-treated culture [26]. However, not all attachment initiates a phage multiplication cycle because phage attachment may be reversible or irreversible [53]. Microscopy of phage attachment may not be suitable for quantifying phage adsorption and indicating reversibility.

In filamentation-associated PAS, some have proposed that the increase in adsorption rate proportional to the increased bacterial surface area corresponded to the increased number of receptors on the bacterial surface [26,52]. Evidence suggests that phages targeting different sites on the same bacteria showed different PAS responses. While the plaque size of phage E79 increased on the bacterial lawn of PA01 in the presence of aztreonam lysine, that of another phage, phiKZ, did not increase even when filamentation was triggered. A possible explanation is that E79 uses LPS as the receptor, whereas phiKZ targets type IV pili (T4P). However, LPS and T4P receptor densities were not well-quantified in that study.

LPS density was reported to be consistent on *E.* coli B/r outer membranes regardless of cell sizes. This was surmised from the proportional increase in the irreversible phage adsorption rate and surface area [52]. A reduction in T4P function in filamented *P. aeruginosa* cells was reported [26], but whether this was related to a reduced surface density of T4P was unclear.

Changes in the bacterial cell wall receptors during filamentation are important because they affect PAS. However, limited investigations have been conducted on phage receptor densities during filamentation.

#### 2.2.2. Accelerated/Delayed Cell Lysis

Cell lysis is the last stage in the infection cycle of lytic phages (Figure 1). The period between phage attachment and cell lysis is called the latent period (Figure 2) [31]. Accelerated cell lysis, or the reduction in the bacterial latent period, promotes a rapid spread of phages in the medium. Hence, the latent time is inversely proportional to phage production [31]. The timing of lysis and the duration of the latent period are determined by phage lysis genes and their resulting protein products during biosynthesis [54]. Lysis genes vary among phages. The canonical lytic system is composed of holin and endolysin and accessory proteins. Holin creates channels in the inner membrane and allows lysin to enter the channel and cleave bonds in the peptidoglycan matrix, which in turn leads to bacteriolysis [55]. Holin concentration, in particular, has been found to be highly relevant to bacterial cell lysis time [23,54]. The absence of the holin gene results in a notably delayed lysis, accompanied by increased burst size in *E. coli* bacteria cells [23,27].

Accelerated bacterial lysis has been reported to be accompanied by PAS in many studies. The addition of 0.030 µg/mL cefotaxime reduced the latent period of T4 and two other T4-type phages (RB33 and RB49) from 2 h to 75–90 min in *E. coli* AS19 [23]. Moreover, cefotaxime (at the tested concentration, 4 × MIC, 0.0625 µg/mL) lessened the latent period of T4 from 24 min to 18 min in *E. coli* ATCC 11303 [56]. The latent period of phage MR-5 in *S. aureus* ATCC 43300 decreased from 33.3 min to 16.6, 18.3, 23.4, and 23.4 min in the presence of sublethal 1 μg/mL linezolid, 0.25 μg/mL tetracycline, 4 μg/mL clarithromycin, and 4 μg/mL telithromycin, respectively [29]. Aztreonam lysine (1.06 μg/mL) shortened the latent period of phage E79 in *P. aeruginosa* PA01 from around 9 to 6 min [26].

Bacterial filamentation is an intermediate state where bacterial chromosomes continuously replicate without cell division [39]. Hence, it is believed that filamented bacteria cells are enriched with more viral components, including holin, than normal cells [27].

Further confirmation of holin expression was measured using engineered T4 phage with an enhanced green fluorescent gene in filamented *E. coli*. A 2.1-fold increase in green fluorescence was detected using a fluorometer [27]. In addition to the increased holin synthesis, it was proposed that the presence of β-lactam antibiotics destabilises bacterial cell walls and reduces the time required for phages to penetrate and degrade bacteria [23,26].

Although holin is increased in filamented cells, its concentration depends on not only the protein expression rate but also the cell dimensions [52]. Conflicting with the abovementioned results, PAS was reported with a delayed lysis observed in T4 phages against *E. coli* K-12 strain with subinhibitory concentrations of cefotaxime and ciprofloxacin, which was due to a decreased holin concentration in the filamented bacterial cell [27]. While Kim et al. [27] detected a 2.1-fold increase in holin expression in filamented cells, the bacteria cell length was increased by 15.4-fold, which greatly increased the cellular volume. As a result, the holin concentration was reduced, and cell lysis delayed [27].

Both accelerated and delayed lysis have been found to be associated with PAS. A shortened latent period or accelerated lysis time increases the rate of repeating the infective cycle, while delayed lysis may allow more time for phage assembly and consequently enlarges the phage burst size [31].

#### 2.2.3. Increased Single Burst Size

The burst size (Figure 2) refers to the average number of phages released per cell after a single infection cycle [23]. Increased phage burst size contributes directly to phage plaque enlargement and has been reported in many studies [23,26,27,29,52,57,58].

Hadas et al. [52] compared the burst size of normal cells with filamented *E. coli* B/r cells induced by pre-treatment with a low concentration of penicillin. Compared with the normal non-treated cells, the filamented cells increased around 4-fold in size along with a 2.5- to 4-fold increase in single burst sizes, suggesting a correlation between bacterial cell size and burst size. Hadas et al. [52] proposed that the burst size depended on the host protein synthesis system, which should increase with the bacterial cell size, but direct measurement of proteins was not conducted.

Kim et al. [27] also observed around a 13-fold increase in burst size in filamented *E. coli* K-12 cells induced by sublethal ciprofloxacin treatment. They postulated that the increased burst size was related to the increased availability of viral components. Further quantification of mRNAs, DNA, and proteins was conducted in these filamented *E. coli* cells, showing a 2-fold increase in the mRNA that encodes T4 phage DNA polymerase, which resulted in also a 2-fold increase in the T4 phage DNA. In addition, a 1.5-fold increase was observed in the mRNA that encodes the phage major capsid protein, but no increase was detected in the capsid protein production. Thus, not all viral components (e.g., capsid proteins) are necessarily increased in the filamented cells. Overall, the relationship between the increased bacterial cell size and increased viral components or other factors is still not well established.

Another hypothesis is that delayed cell lysis (see Section 2.2.2) allows a longer time for phage biosynthesis and maturation [23,52]. Hadas et al. [52] delayed the timing of lysis by superinfection with phage T4 (where the secondary adsorption of additional T4 phage leads to lysis inhibition, hence delaying the lysis time [59,60]) in penicillin-pre-treated *E. coli* culture from 20 min to 28 min. The single burst size was enhanced from 270 to 700 plaque-forming units per cell [52]. Kim et al. [27] reported 2.5-fold and 10.5-fold increases in the burst size of T4 accompanied by a 5 min delay in lysis time, with sublethal concentrations of cefotaxime and ciprofloxacin, respectively. A correlation between delayed cell lysis time and an increase in burst size of phage T4 was supported by these data.

The increased burst size is one of the contributors to PAS, but it is not the only factor determining the overall phage production. Other factors, such as the latent period, should also be considered for PAS investigation.

### 2.3. PAS with Temperate Phages

Other than filamentation-induced PAS in lytic phages, a recent in vitro study also reported temperate phage–antibiotic synergy, so-called “tPAS” [61]. Temperate phages infect hosts by integrating the genetic material of the phages into the bacterial chromosome. The phage genome replicates along with bacteria replication. Phages do not release until triggered by a stimulus. The temperate phage genome-incorporated chromosome is called prophage, and the process is called lysogeny or the lysogenic cycle. The bacteria that carry prophages are called lysogens.

Al-Anany et al. [61] examined temperate phage HK97 (tested range: MOI from 0 to 50) with ciprofloxacin (from 0 to 100 ng/mL) against host *E.coli* K-12. Eradication (≥8 log reduction) was achieved by HK97 (MOI ≥ 10) with either MIC or ½ × MIC ciprofloxacin after overnight incubation, whereas HK97 alone was ineffective against *E. coli* K-12. The study suggests that the latent period and burst size were not affected by the addition of ciprofloxacin. One of the underlying mechanisms was improved prophage induction triggered by ciprofloxacin via the *recA*-mediated SOS response. As mentioned in Section 2.1, RecA proteins facilitate the cleavage of many phage repressors and can lead to improved prophage induction [47,48]. This mechanism was confirmed using mutant strain BW25113 Δ*recA* with gene *recA* deletion, which did not exhibit synergy with ciprofloxacin [61]. Another underlying mechanism was the increased sensitivity of lysogens to ciprofloxacin. The MIC of ciprofloxacin on lysogens was 4-fold lower than that of wild-type bacteria, but the reason is unknown [61].

The antimicrobial effects achieved by HK97 with a sublethal concentration of ciprofloxacin are significant. However, “tPAS” is as of yet poorly studied. More investigations with other bacteria species with in vitro and in vivo models are needed for implementing its use therapeutically.

### 2.4. Limitations in Assessing PAS

PAS has been assessed by bacterial filamentation and plaque size enlargement; however, these effects may not always occur. The PAS response was found in *P. aeruginosa* phages with ciprofloxacin in the absence of bacterial filamentation [27]. Moreover, synergy screening between *P. aeruginosa* phage PA22 with tetracycline showed no significant plaque size enlargement even when bacterial filamentation occurred [27].

In another study tested with ampicillin, penicillin G, kanamycin, rifampicin, and tetracycline, tetracycline was found to produce the greatest plaque size increase, yet the bacterial cell size increase was the smallest [62], suggesting that increased bacteria cell size and surface area are not the determinants of phage replication. However, due to the lack of information on the phage adsorption rate, latent period, and burst size in these studies, it cannot be confirmed whether antibiotics triggered PAS through routes other than filamentation.

Due to the large number of available antibiotics and the variations between different phages and bacterial hosts, it is very difficult and time-consuming to systematically assess the PAS response as well as the corresponding characteristics of the phage lytic cycle. This has hindered researchers in further exploring the underlying mechanisms.

## 3. The Development of Bacterial Resistance to Phages and Antibiotics

To date, there are various marketed antibiotics for treating bacterial infections. However, after prolonged exposure to the same or similar antimicrobial agents, bacteria can evolve and develop resistance by reducing drug uptake, increasing drug efflux, modifying drug target sites, and inactivating the drug [63,64].

Various approaches to using phages and antibiotics, with different mechanisms of inhibiting MDR infections, have been widely investigated. While a single-agent treatment kills its sensitive population and selects resistant bacteria, treatments with two or more agents with different mechanisms of action cover larger host populations. For instance, the combined use of phages and antibiotics leads to the simultaneous selection of phage-sensitive and antibiotic-sensitive populations, resulting in a very small or non-existent population resistant to both agents [22].

In addition, with lethal concentrations of antibiotics and phages, bacteria are unlikely to rapidly evolve and acquire resistance to multiple agents that target different pathways in a short period of time. In particular, when the development of resistance to either agent adversely alters the bacterial components/functions involved in the attacking pathways of the other agent, adaptation trade-offs happen [22].

For example, one of the known adaptation trade-offs between antibiotic resistance and phage resistance is multidrug efflux pump development and alleviated phage attachment. Some bacteria developed a resistance to antibiotics by expressing multidrug efflux pumps that remove the drug from the cells, hence the bacteria become multidrug- or pan-drug-resistant [65]. On the other hand, phages are known to attach to various receptors on the bacterial cell surface to initiate infections. Similar to the development of resistance to antibiotics, bacteria can modify surface proteins against phages. This may consequently attenuate the resistance to antibiotics by altering bacterial multidrug efflux pumps.

Several studies observed gene mutations or reduced protein expression of bacterial drug efflux pumps after combined treatment with phages and bacteria [20,21]. This reduced expression of proteins that are responsible for antibiotic resistance is due to the emergence of phage resistance. *P. aeruginosa* phage OMKO1 binds with hosts at the outer membrane porin M (OprM) of the multidrug efflux pump. OprM was knocked out in engineered phage-resistant *P. aeruginosa* strain to force phage resistance. The engineered strain then regained sensitivity to antibiotics such as ciprofloxacin, tetracycline, ceftazidime, and erythromycin. The minimal inhibitory concentrations (MICs) of these antibiotics decreased by 12-fold compared to non-gene-modified strains [19]. The MICs of multiple antibiotic classes were found to decrease after phage mono-treatment against MDR bacteria [19,20]. In a murine lung infection model treated with pulmonary-delivered *Pseudomonas*-targeting phage PEV31, phage-resistant isolates of *P. aeruginosa* regained sensitivity to ciprofloxacin [66,67].

## 4. Applications of Phage–Antibiotic Therapy

### 4.1. Reduced Antibiotic Dose for Phage–Antibiotic Treatment

Phage–antibiotic therapy has promising therapeutic potential for enhancing antimicrobial effects due to PAS and decreasing the likelihood of resistance development. Moreover, it reduces the required antibiotic concentration compared to mono-antibiotic treatment.

Decreased antibiotic MIC when used in combination with phages was observed in several studies [16,68,69,70,71]. For example, combined treatment with *Pseudomonas*-targeting phage PEV20 and ciprofloxacin enhanced *P. aeruginosa* biofilm eradication, highlighting the potential for reducing the antibiotic concentration required to combat highly recalcitrant infections associated with biofilms [68]. Although increasing the antibiotic concentration in phage–antibiotic treatment is expected to increase synergy, in some situations it may result in antagonism. When *P. aeruginosa* biofilms were treated with phage EPA1 and ciprofloxacin, meropenem, and gentamicin, synergism was observed at 1 × MIC with a 4.7, 4.1, and 2.6 log reduction in biofilm density, respectively. However, antagonism of antimicrobial activity was observed in ciprofloxacin and meropenem at 8 × MIC [16]. This is because these antibiotics functioned as protein or DNA synthesis inhibitors to disrupt phage replication [69] (refer to Section 4.2.1).

In contrast, increasing the ciprofloxacin dose from a sublethal dose (1/10 × MIC and 1/5 × MIC) to a lethal dose up to 1 × and 2 × MIC successfully inhibited the regrowth of *E. coli* in combination with phage ELY-1, without compromising the antimicrobial effect [70]. In another study, bacterial regrowth was observed when an anti-*A. baumannii* phage Ab105-2jDCI was combined with meropenem and imipenem at 1/8 × MIC. On the other hand, synergism occurred with no regrowth of bacteria when the antibiotic concentration was increased to ¼ × MIC [71]. Thus, depending on the antibiotic classes and concentrations, low doses may generate bacterial resistance, but high doses may not necessarily produce a proportionally greater antimicrobial effect. Therefore, antibiotic concentrations should be increased moderately when used with phages for treating bacterial infections.

### 4.2. Effect of Administration Time and Sequence on Bacteria Inhibition

#### 4.2.1. Aminoglycosides

Several studies [16,70,72,73] showed that the timing and sequence of administrating phages and antibiotics can impact the killing effects on different bacterial strains. Simultaneous administration of phages and drugs has shown synergistic killing in many studies. However, for some antibiotic classes, sequential instead of simultaneous treatment exerted higher efficacy in inhibiting bacterial growth.

Aminoglycosides, which inhibit protein synthesis, did not show significant synergism with phages in simultaneous administration but showed better bacterial killing and enhanced phage production in sequential administration [72,73,74,75,76].

Torres-Barcelo et al. [73] conducted experiments in which streptomycin (100, 240 μg/mL) was added at 0, 12, and 24 h after *P. aeruginosa* strains were initially treated with phage LUZ7 (10^5^ pfu/mL). Simultaneous treatment strongly suppressed the growth of *P. aeruginosa* with no regrowth over 70 h. The most significant synergistic bactericidal effect (*p* = 0.027) occurred when streptomycin was administered 12 h after phage treatment.

Moreover, co-treatment with an anti-pseudomonal phage cocktail (phage NP1 and phage NP3) combined with gentamicin and tobramycin (8 × MIC) [72] hampered phage replication as the titer was reduced (*p* < 0.04). Synergism occurred when the antibiotics were administered 24 h after pre-treatment with the phage cocktail, showing a significant reduction (from 8 to 2 log) in *P. aeruginosa* biofilm density. In another study, two aminoglycosides (kanamycin and neomycin) were compared with another protein synthesis inhibitor antibiotic, tetracycline, in co-treatment with phage T3 on *E. coli* biofilm. Interestingly, tetracycline showed no inhibition of phage infectivity even though it has a similar antibacterial mechanism (inhibiting protein synthesis) to kanamycin and neomycin, which decreased the phage burst size, replication, and efficiency of plating [74]. This antagonistic effect may be caused by the additional function of aminoglycosides on the mistranslation of the phage protein, as they are known to mistranslate coat proteins, lysosomes, and maturation proteins.

Aminoglycosides led to a misreading of different cistrons or genes on the 30 s ribosomal subunit, which eventually inhibited the initiation of different proteins. For example, kanamycin and gentamicin inhibited the translation of coat proteins, whereas kasugamycin inhibited maturation proteins [75]. However, the actual mechanism of action of aminoglycosides needs further investigation because the downstream signalling system is sophisticated. It has been shown that the antagonism by kanamycin and streptomycin occurred after injection of phage genomes into host cells, resulting in the inhibition of phage proliferation [76]. Phage production can be easily interrupted by the inhibition of protein synthesis by aminoglycosides. Therefore, a sequential approach should be considered; it allows phages to replicate effectively and avoid the disruption of the assembly process.

Based on the above findings, a delay in the administration of antibiotics (about 6–12 h) after phage pre-treatment led to stronger antimicrobial activity; this observation was more prevalent with aminoglycosides than other antibiotic classes [74]. To maximise the therapeutic antimicrobial effect of phage–antibiotic therapy, the administration time and sequence of the phages and antibiotics need to be optimised. To achieve that, further investigations are needed for a better understanding of phage–antibiotic interactions regarding the dynamics of antibiotic concentration as well as phage and bacteria titer over time.

#### 4.2.2. Ciprofloxacin and Tetracycline

DNA synthesis inhibitor ciprofloxacin seemed to yield contradictory results, as a phage–ciprofloxacin combination can exert similar killing effects regardless of the administration time. Both simultaneous and sequential treatment (antibiotics administered after phages) exerted synergism against bacteria *P. aeruginosa*, *E. coli*, and *Salmonella typhimurium*.

When ciprofloxacin (1 × MIC) was given simultaneously with phage PA14, a synergistic effect against *P. aeruginosa* was observed despite titer reduction (1.8 log) as compared with sequential treatment, although it was not statistically significant (*p* > 0.05) [72]. This decrease in phage titer may be due to the mechanism of ciprofloxacin in inhibiting DNA replication by interfering with the host cell DNA gyrase, which is responsible for relaxing the DNA supercoils during DNA synthesis. DNA gyrase is also involved in phage genome replication. It was found that the inhibition of host DNA gyrase by antibiotics reduced phage replication, regardless of whether the phages carry their own DNA gyrase gene. Quinolone antibiotics, such as nalidixic acid and ciprofloxacin, act as inhibitors of this enzyme and decreased 50% of the phage burst size compared to control at a low concentration of only 0.3 mg/mL against *E. coli* [77]. However, different results were shown in the study by Lopes et al. [70]. Ciprofloxacin was added after 0, 6, 12, and 18 h of phage ELY-1 exposure on *E. coli*. The bacterial killing was maximised when ciprofloxacin (1 × MIC) was added 6 h after the phage pre-exposure. Bacterial density was significantly reduced (2.3 log; results measured by bioluminescent assay) as compared to other administration times (0, 12, and 18 h), with no bacterial regrowth observed at the end of the experiment. The phage titer in all delayed ciprofloxacin treatments increased significantly (1.7–2.0 log) compared to simultaneous administration. These results agreed with those from another study [78], in which *S. typhimurium* pre-treated with the phage for 6 h and followed by ciprofloxacin showed the highest reduction (3 log) in bacteria count as compared with using antibiotics or phages alone. Contradictory results were observed with tetracycline, where simultaneous treatment with phages showed antagonism in *S. aureus* [79] but not *E. coli* [74], showing that the PAS effect may not be generalisable, but rather specific to the bacteria, phages, and antibiotic classes involved. The latter plays a critical role in phage–antibiotic treatment, as the mechanism of action varies according to the antibiotic class. Even with some antibiotics with similar mechanisms, their combined bactericidal effects with phages can be completely distinct from each other. Thus, the decision to apply simultaneous or sequential treatment depends on the mechanism of action of the antibiotic, which can affect different parameters, such as phage burst size and plaque size. A well-designed and structured method is important.

#### 4.2.3. The Impact of Host Strains in Sequential Treatment and Host Environment on the Efficacy of PAS Treatment

Recently, Wang et al. [80] indicated that the killing effect of combined treatment not only depends on the sequence of antibiotics and the administration time but also on the host strains. Host strains acquired clinically can be multi-drug-resistant compared to laboratory strains. Partially resistant strains may exert the critical phage–antibiotic trade-off mechanism mentioned above (Section 3).

Phage Sb-1 and an antibiotic (doxycycline, levofloxacin, linezolid, clindamycin, or rifampin) were used together on an *S. aureus* reference strain and five rifampin-resistant *S. aureus* (RRSA) strains, three of which were methicillin-susceptible and two were methicillin-resistant. Synergistic effects on biofilm reduction were compared between simultaneous and staggered administrations of the antibiotics and phage. Most strains were susceptible to the antibiotics, whereas two strains showed resistance only to doxycycline and levofloxacin. No synergistic effects were observed in the inhibition of anti-biofilm activity in any of the combined treatments with simultaneous application (10^6^ PFU/mL, 64–256 µg/mL) on all the strains, except the phage–rifampin combination on the reference strain. However, in sequential treatment, synergism was observed in doxycycline (16–128 µg/mL) and linezolid (128, 256 µg/mL) after 24 h of phage (10^6^ PFU/mL) pre-exposure. Biofilm density (measured by isothermal microcalorimetry) was strongly reduced in all six strains, including the resistant strains, by the phage–levofloxacin (16–128 µg/mL) and phage–clindamycin (64–256 µg/mL) treatments, with five and four strains, respectively, out of the six showing synergistic effects on reducing biofilm density. Rifampin was the only exception; the combination therapy had no bactericidal synergism in any RRSA strain. Rifampin was the only antibiotic that showed minimal antimicrobial activity in all RRSAs, and this was due to the high resistance of the clinical strain, as the binding site of rifampin was altered by the mutation of *rpoB* on RNA polymerase. The binding affinity of rifampin to bacterial RNA polymerase was reduced [81].

Although staggered administration is an essential factor in exerting synergism, the host strain is also important. Synergism was observed in some RRSA strains but not others, showing that biofilm inhibitory effects were different even when using the same phage–antibiotic (levofloxacin) combinations. The above results demonstrated that host strains can show different inhibition profiles and synergistic effects [82], but these findings were limited to the number of host strains examined, as not all host strains showed different inhibitory effects when the same combined treatment was given. Nevertheless, the majority of phages are highly specific to the bacterial strain, as bacteria may not be sensitive enough to all phages to allow synergism to occur.

These studies only demonstrated the phage–antibiotic treatment on a single bacterial species. When phage EPA1 and gentamicin were used on biofilms of dual bacterial species, *P. aeruginosa* and *S. aureus* [16], no synergism was observed in simultaneous treatment on either species. In comparison, sequential treatment resulted in an approximately 6.3 log reduction on *P. aeruginosa* biofilms, whereas *S. aureus* biofilms only had a 2 log decrease. This was due to the specificity of the phage, as EPA1 is an anti-pseudomonal phage. The reduction in *S. aureus* biofilms was caused by the administration of a high concentration of gentamicin (8 × MIC).

As the pharmacokinetics of antibiotics in physiological media such as serum and urine can vary, phage–antibiotic efficiency is altered accordingly. Treating *E. coli* infection with phage ϕHP3 and ceftazidime in human pooled urine and heat-inactivated serum showed completely different results compared to that in Luria-Bertani (LB) medium [83]. The antagonism of bacteria in serum occurred in the entire tested concentration range of ceftazidime, except at the highest concentration (256 µg/mL) with 10^9^ PFU/mL of phage, which showed an additive effect. Similar results were found in urine in which only high doses of phage (10^9^ PFU/mL) and ceftazidime (256 µg/mL) produced synergism. In contrast, synergism was observed in LB medium at low doses of ceftazidime (2 µg/mL) and phage (10^4^ PFU/mL). The overall reduction in the bacterial level in serum and urine may not be due to the high doses, but the low starting bacterial inoculum, as untreated bacteria gradually decrease in the presence of urine and serum. This phenomenon was also observed in another study with *E. coli* isolates in urine samples [84]: bacterial growth was significantly lower in concentrated urine than in a diluted urine sample (*p* < 0.001). It was found that urea was the main factor for reduced *E. coli* growth. Increased urea concentration corresponds to reduced bacteria count [84].

Overall, due to the specificity of phages and the diverse pharmacokinetics of antibiotics, phage–antibiotic efficiency depends on the host strains and physiological media.

### 4.3. In Vivo Efficiency of Phage and Antibiotic Therapy

Although phage–antibiotic treatment is widely studied in vitro, little research focuses on preclinical in vivo trials to evaluate the efficacy of combination treatment. Promising results in treating bacterial infection showed the potential of phage–antibiotic treatment in vivo (Table 1). Mice and *Galleria mellonella* were extensively used for testing a wide range of bacterial infections.

Mitomycin C and imipenem were combined with lytic phage vB_KpnM-VAC13 to treat imipenem-resistant strain K2534 and persisted strain K3325 of Gram-negative bacteria *K. pneumoniae* in larvae of moth *Galleria mellonella*. The survival rate of the larvae significantly increased to 50% and 75% when co-treated with phage and mitomycin C or imipenem, respectively, compared to either antibiotic or phage monotherapy, except phage–imipenem co-treatment of the imipenem-resistant strain. This was due to the hydrolysis of imipenem by β-lactamase in the resistant strain. Nonetheless, the mortality rate of the larvae significantly decreased with all other combined treatments [85]. Additionally, the emergence of a resistant mutated strain was not detected in combined treatment. Similar results were shown in other *Galleria mellonella* models, achieving a 75% survival rate in treating *E. coli* infection when phage ΦWL-3 was used in combination with fosfomycin [82]. In addition, phage in combination with meropenem and imipenem against *A. baumannii* increased the survival rate by approximately 30% (*p* < 0.05) compared to phage monotherapy [71]. Utilisation of the *Galleria mellonella* model has enabled high numbers of replicates, but their physiological characteristics are much different to those of mammals, so rodent infection models would be more relevant.

A neutropenic mouse model was used to determine the efficacy of phage PEV20 combined with ciprofloxacin on *P. aeruginosa* lung infection. Co-spray dried phage and ciprofloxacin powder administrated intratracheally significantly reduced the bacterial density compared to single treatment with either agent [25]. In addition, in a rat model with endocarditis induced by *P. aeruginosa*, a phage cocktail combined with ciprofloxacin was highly synergistic, resulting in negative vegetation in 7 out of 11 rats with more than a 6 log reduction in bacterial concentration, as opposed to zero rats with either phage or ciprofloxacin monotherapy [86]. Bacterial concentration was inversely correlated with phage titer. Mouse locomotor activity, lesion, oedema level, and bacterial burden were determined by infecting the methicillin-resistant *S. aureus* (MRSA) ATTC 43300 strain to induce prosthetic joint and diabetic foot infections [87,88]. The combination of phages MR-5 and MR-10 with linezolid significantly reduced the level of lesion, oedema, and bacterial burden by 4.5 log and 5 log, respectively, as well as maximising locomotor activity caused by MRSA compared to the untreated control.

These findings on animal studies of phage–antibiotic combined treatments demonstrated a reduction in bacterial burden caused by both Gram-positive and Gram-negative bacteria, and the ability to recover from the infections increased considerably.

### 4.4. Clinical Case Studies

Phage therapy was applied to patients in the early years of its discovery, but its use was decreased due to the lack of regulation or standardised methodology, and for safety reasons [7]. In view of phage–antibiotic administration showing promising antimicrobial effects in vitro and in vivo, and given the current antibiotic resistance crisis, personalised combination therapy has been studied in individual patients.

A phage cocktail that consisted of six lytic phages combined with trimethoprim-sulfamethoxazole was used to treat a recurrent urinary tract infection with drug-resistant *K. pneumoniae* in a 63-year-old female with Type-2 diabetes and hypertension. Phage monotherapy failed twice, as phage resistance caused re-emergent bacterial isolates. When trimethoprim-sulfamethoxazole was administered orally twice a day with the phage cocktail once a day through bladder irrigation, the resistant strain was completely inhibited, and the urinary tract infection did not recur 6 months after discharge from hospital [8].

However, not all patients with chronic infections had the same recovery profile after treatment. An 86-year-old female patient with chronic obstructive pulmonary disease was infected by carbapenem-resistant *A. baumannii* (CRAB). Phage Ab_SZ3 was administrated with tigecycline and polymyxin E. The phage was given by a vibrating mesh nebuliser once daily, with gradually increasing doses from 5 × 10^6^ PFU to 5 × 10^10^ PFU on Day 13. Tigecycline was given intravenously for the first 5 days of treatment, followed by oral polymyxin E for another 5 days. In a total of 16 days of treatment, no CRAB cultures were detected in the patient’s sputum from the 7th day onwards, with only one exception on the 15th day, when a positive CRAB culture was found. One month after phage treatment, the patient developed sepsis caused by *E. faecium* and *S. haemolyticus*. During treatment with vancomycin, colonisation by *P. aeruginosa* was also found in the patient’s bronchoalveolar lavage fluid (BALF) culture. Even with the occurrence of different types of bacterial infection and the pressure of antibiotic use, CRAB showed no reappearance after the combination treatment [89].

As an intact immune system plays an important role in suppressing bacterial infections with opportunistic pathogens [90], chronic infections are more prevalent in immunocompromised patients. The recurrence of bacterial infections of different resistant species are often seen after the initial treatment. Hence, complete eradication is needed to reduce relapses of bacterial infections in immunocompromised patients.

In a pediatric patient with cystic fibrosis female infected with pan-drug-resistant *Achromobacter* species, the combination therapy of phage Ax2CJ45f2 and two antibiotics (cefiderocol and meropenem/vaborbactam) by intravenous administration improved forced expiratory volume in one second (FEV_1_, an indicator of lung function) from 33% to 60%. The symptoms were alleviated with decreased sputum production and reduced cough. No adverse effects were observed with the combined treatment [91].

Above all, phage–antibiotic combinations have produced encouraging outcomes in treating multi-drug-resistant bacterial infections. However, standardisation of the treatment method is difficult, as patients have different medical histories and varying levels of immunity. The administration route, time, and dose of phage and antibiotics differed for each patient. Formulating personalised treatments is time-consuming and expensive, but standardised regimens may not exert the same efficiency in all patients.

## 5. Challenges

### 5.1. Polymicrobial System and Biofilm

While most studies focused on the synergistic effects of phages and antibiotics against one bacterial strain or species, there is a demand for investigations of the phage–antibiotic treatment of polymicrobial systems. In clinical settings, bacterial infections are usually associated with multiple strains or species. As a result, the genetic pool is enlarged in these polymicrobial systems, and there are more opportunities for mutations to occur in bacteria as well as phages. These uncertainties may produce varying efficacies in phage–antibiotic therapy.

Another challenge of polymicrobial systems is the typical formation of biofilm. The spatial structure of biofilm may prevent antimicrobials from reaching sensitive populations. Hence, their efficacy against biofilms is reduced compared to planktonic cells. Biofilms also allow more time for co-evolution between phage- and antibiotic-selected populations under prolonged exposure to phage and antibiotics. For instance, an in vitro study reported that co-administration of *P. aeruginosa* phage 14/1 with gentamycin reduced bacteria density in biofilm for the first 12 days, but then regrowth occurred due to resistance development [92].

### 5.2. Mutation Dynamics of Bacteria and Phages in the Presence of Anitbiotics

It is believed that phages have the advantage of co-adapting with bacteria; hence, they can overcome the development of bacteria resistance to phages in recurrent or chronic infections. However, the mutation rate of bacteria *P. fluorescens* SBW25 was found to be faster than that of bacteriophage SBW25_Φ_2 [93].

Furthermore, Cairns et al. reported that the bacterial density of *P. fluorescens* SBW25 was the lowest after 66-days of co-treatment with SBW25_Φ_2 (MOI = 0.01) and streptomycin (2 µg/mL, 1/10 × MIC) compared to SBW25_Φ_2 with streptomycin at 0.2 µg/mL or without streptomycin. The phage titer decreased more significantly with a higher concentration of streptomycin, and no phage was detected with 2 µg/mL (1/10 × MIC) streptomycin by Day 14, whereas only a small decrease of around 1 log in phage titer was observed on Day 66 without streptomycin. The phage titer started to decline as bacteria hosts developed resistance to the phage [94]. Another overnight experiment further correlated the addition of streptomycin with the development of bacterial resistance to phage. The mutation rate was 121, 179, and 167 mutation events/mL for 0, 0.2, and 2 µg/mL, respectively (*p* < 0.001 for 0.2 and 2 µg/mL streptomycin compared with no streptomycin. No statistical significance was detected between streptomycin treatments of different concentrations) [94]. This observation denied the hypothesis that the combined treatment with phage and antibiotics reduces the mutation rate of bacteria due to the fitness costs associated with resistance development [22].

As there is only limited research that investigated the long-term mutation dynamics of bacteria and phages in the presence of antibiotics, it is unknown whether the presence of antibiotics promotes the mutation rate of bacteria and/or phages, or whether the changes in mutation rates vary depending on the bacteria species, antibiotic class, or phage type.

### 5.3. Phage–Antibiotic Pharmaceutics Development

So far, phage-based clinical therapies are mainly personalised. Unlike antibiotics, the specificity of phage has hindered its wide use as predefined formulations on the market. In response to that, strategies commonly considered include the modification of the antimicrobial spectrum of phages through genetic engineering [95], the expansion of phage banks, and seeking phages for different targeted receptors and hosts to establish the broadest possible coverage. The use of complementary phage cocktails with antibiotics [20,96,97,98] is an attractive strategy, and progress has been made in the development of phage and antibiotic formulations. Formulations such as the PEV20-ciprofloxacin inhalable formulations for use against *P. aeruginosa* lung infection [24,99] and hydrogel membrane for the topical delivery of minocycline and phages [98] have shown promising in vitro and in vivo antimicrobial performance [25,98]. Still, there is a long road ahead towards a successful phage-based pharmaceutical product. We still lack information on their optimal dosage, “pharmaco- and phage-kinetics”, long-term stability, storage, and quality control measures [100,101].

## 6. Conclusions and Future Perspectives

As antibiotic resistance is a life-threatening danger to global health, phage therapy has attracted attention over the past few decades as a potentially efficient alternative solution. The abundance, specificity, and versatility of phages are their advantages. Since antibiotics have been found to enhance phage production and further increase antimicrobial activity, phage–antibiotic combination treatments have been extensively studied. In the presence of antibiotics, instead of dividing, bacteria undergo elongation (filamentation), which facilitates phage adsorption, modifies bacterial cell lysis time, and/or increases phage burst size, which in turn results in enhanced phage replication. In vitro experiments showed synergistic antimicrobial effects with phage–antibiotic co-treatment, resulting in a significant reduction in bacterial growth. Moreover, antibiotic doses and administration sequence are two factors contributing to the variability in synergism. High antibiotic doses do not necessarily produce better bactericidal effects with phages, as inhibition driven by antibiotics may adversely affect phage production. The antibiotic suppression of phage replication in simultaneous administration was demonstrated in several studies, while sequential treatment with phages followed by antibiotics showed better antibacterial effects. The order of administration depends on the specific antibiotic. In vivo efficiency generally agreed with in vitro findings. Promising outcomes with better survival and faster healing have been presented in rodent models and larvae models when combination therapy was given. Further investigations with other animal models or systems need to be developed for a better understanding of phage and antibiotic co-therapy. In clinical case studies, phages combined with antibiotics improved treatment outcomes and reduced bacterial growth. Nevertheless, recurrent bacterial infections in immunocompromised patients are difficult to treat.

Successful clinical cases of personalised phage–antibiotic treatment in tackling persistent MDR infections have been reported, but much progress needs to be made before it becomes popular. Firstly, as phages are bacteria-specific, a rapid diagnosis should be developed for precise phage identification. Secondly, world-wide phage banks are needed for the quick selection of suitable phages. Thirdly, dose regimens, including dosage and administration sequence, must be optimised for safe and efficient phage–antibiotic treatment for infections of different severity. Current or completed clinical trials on bacteriophages lack data for their combined use with sublethal dose of antibiotics. Although phage with sublethal doses of antibiotic reported more benefits than those with lethal doses of antibiotics in in vitro models, it may be not ethical for clinicians to prescribe sublethal doses of antibiotics based on existing clinical trials. Hence, further dose optimisation and clinical trials are needed. Fourthly, to prevent a regrowth of uncleared MDR bacteria, researchers should develop a strategy or dose regimen to achieve full pathogen eradication. In addition, phage–antibiotic therapy in association with innate immune system responses is another possible and promising method for eradicating MDR infections. Finally, rapidly formulating methods must be developed for improved delivery to sites of infections.

While individualised treatment is the current mainstream of phage-based therapy, it is time-consuming and expensive. Predetermined phage–antibiotic formulations are considered for improved cost-effectiveness, but selecting suitable phage–antibiotic combinations is another challenge because they are not always synergistic against all bacterial strains. Extensive foundational work in synergy screening is needed for selecting phage–antibiotic combinations against different species. Moreover, formulating methods must be developed or optimised for improving stability for convenient storage and transport, and for adjusting the release of phages and antibiotics to achieve either simultaneous or sequential treatments. We believe that with increasing evidence from in vitro and in vivo works, as well as future clinical trials, it is feasible to develop predetermined formulations.

Current methods in treating bacterial infections are not necessarily limited to conventional phage–antibiotic combined treatments; genetically engineered phages and phage-coded enzymes have also attracted recent attention. By modifying the tail fibre genes of phages to increase the host range for multiple targeting, the bactericidal effects would be significantly enhanced. Endolysin is an example of a phage-coded enzyme. Its rapid and potent killing of bacteria without apparent resistance development is attractive and may become a potential treatment for MDR infections. By combining new techniques and agents with antibiotics, phage–antibiotic combined treatment is clearly more promising for treating resistant bacterial infections than mono-phage therapy.

## Figures and Tables

**Table 1:** In vivo study of phage–antibiotic treatment in bacterial killing.

Animal Model	Bacterial Species	Drugs Used	Phage	Highlights
Moth larvae (*Galleria mellonella)* [85]	*Klebsiella pneumoniae*	Mitomycin C, imipenem (1/4 × MIC, 1/2 × MIC)	vB_KpnM-VAC13 (10^7^ or 10^9^ PFU/mL)	Survival rate of larvae significantly increased to 50% and 75% when phage co-treated with mitomycin C and imipenem, respectively, in treating resistant strain and persisted strain, compared to either monotherapy, except for phage/imipenem on resistant strain.
Moth larvae (*Galleria mellonella)* [71]	*Acinetobacter baumannii*	Imipenem, meropenem (1/4 × MIC, 1/8 × MIC)	Ab105-2φ∆CI (10^8^ PFU/mL)	Combination therapy and meropenem alone had same survival rate; both survival rates were higher than phage monotreatment (*p* < 0.05); imipenem combined with phage showed high survival rate compared to monotherapy (*p* < 0.05).
Mouse: lung infection [25]	*Pseudomonas aeruginosa*	Ciprofloxacin (0.33 mg/mg)	PEV20 (10^6^ PFU/mg)	PEV20 combined with ciprofloxacin significantly decreased bacterial concentration by 5.9 log, where either monotherapy showed no obvious bacterial reduction.
Moth larvae (*Galleria mellonella)* [82]	*Escherichia coli*	Fosfomycin (200 mg/kg)	fWL-3 (10^7^ PFU)	Simultaneous treatment with phage and fosfomycin had higher survival rate than sequential treatment in both EC1 and ATCC 25922 strains. Phage and fosfomycin showed 75% of survival rate in ATCC 25922 strain.
Rat [86]	*Pseudomonas aeruginosa*	Ciprofloxacin (0.19 μg/mL)	Phage cocktail PP1131 Bolus injection (1 mL of 10^10^ PFU/mL in 1 min) Continuous infusion (0.1 mL/h of 10^10^ PFU/mL over 24 h)	Phage/ciprofloxacin exerted highest synergistic effects with 6 log bacterial reduction and achieved 64% reduction in bacterial infection. No phage-resistant mutants in vivo.
Mouse: prosthetic joint infection [87]	Methicillin-resistant *Staphylococcus aureus*	Linezolid (5% *w*/*w*)	MR-4 (10^9^ PFU/mL)	The combined treatment with phage and linezolid maximised the mice locomotor activity, reduced oedema at the affected limb, and significantly reduced the bacterial burden (~4.5 log) as compared with the untreated control.
Mouse: diabetic foot infection [88]	Methicillin-resistant *Staphylococcus aureus*	Linezolid (25 mg/kg)	MR-10 (10^8^ PFU/mL)	The combination of phage and linezolid demonstrated a high antimicrobial effect in reducing the bacterial load (5 log) and lesion level. Healing was accelerated at Day 7 after the co-treatment compared to the untreated control (Day 12).

## Data Availability

Not applicable.

## Abbreviations

BALF, bronchoalveolar lavage fluid; CRAB, carbapenem-resistant *A. baumannii*; CRISPR, clustered regularly interspaced short palindromic repeats; FEV_1_, forced expiratory volume in one second; LB, Luria-Bertani; LPS, lipopolysaccharides; MDR, multidrug-resistant; MIC, minimal inhibitory concentration; MRSA, Methicillin-resistant *Staphylococcus* aureus; OprM, outer membrane porin M; PAS, phage–antibiotic synergy; PBP, penicillin-binding proteins; PFU, plaque-forming unit; RRSA, rifampin-resistant *S. aureus*; T4P, type 4 pili; TEM, transmission electron microscope.

## References

[1] Rossolini G.M., Arena F., Pecile P., Pollini S. (2014). Update on the antibiotic resistance crisis. Curr. Opin. Pharmacol..

[2] De-Oliveira D.M.P., Forde B.M., Kidd T.J., Harris P.N.A., Schembri M.A., Beatson S.A., Paterson D.L., Walker M.J. (2020). Antimicrobial resistance in ESKAPE pathogens. Clin. Microbiol. Rev..

[3] Global Priority List of Antibiotic-Resistant Bacteria to Guide Research, Discovery, and Development of New Antibiotics. https://www.who.int/medicines/publications/WHO-PPL-Short_Summary_25Feb-ET_NM_WHO.pdf.

[4] (2019). Antibiotic Resistance Threats in the United States. https://stacks.cdc.gov/view/cdc/82532.

[5] Chang R.Y.K., Wallin M., Lin Y., Leung S.S.Y., Wang H., Morales S., Chan H.K. (2018). Phage therapy for respiratory infections. Adv. Drug Deliv. Rev..

[6] Housby J.N., Mann N.H. (2009). Phage therapy. Drug Discov. Today.

[7] Eaton M.D., Bayne-Jones S. (1934). Bacteriophage therapy—review of the principles and results of the use of bacteriophage in the treatment of infections. JAMA.

[8] Bao J., Wu N., Zeng Y., Chen L., Li L., Yang L., Zhang Y., Guo M., Li L., Li J. (2020). Non-active antibiotic and bacteriophage synergism to successfully treat recurrent urinary tract infection caused by extensively drug-resistant *Klebsiella pneumoniae*. Emerg. Microbes Infect..

[9] Dedrick R.M., Guerrero-Bustamante C.A., Garlena R.A., Russell D.A., Ford K., Harris K., Gilmour K.C., Soothill J., Jacobs-Sera D., Schooley R.T. (2019). Engineered bacteriophages for treatment of a patient with a disseminated drug-resistant *Mycobacterium abscessus*. Nat. Med..

[10] Bertozzi Silva J., Storms Z., Sauvageau D. (2016). Host receptors for bacteriophage adsorption. FEMS Microbiol. Lett..

[11] Hyman P., Abedon S.T. (2010). Bacteriophage Host Range and Bacterial Resistance. Adv. Appl. Microbiol..

[12] Altamirano F.L.G., Barr J.J. (2019). Phage therapy in the postantibiotic era. Clin. Microbiol. Rev..

[13] Labrie S.J., Samson J.E., Moineau S. (2010). Bacteriophage resistance mechanisms. Nat. Rev. Microbiol..

[14] Leon M., Bastias R. (2015). Virulence reduction in bacteriophage resistant bacteria. Front. Microbiol..

[15] Vasu K., Nagaraja V. (2013). Diverse functions of restriction-modification systems in addition to cellular defense. Microbiol. Mol. Biol. Rev..

[16] Akturk E., Oliveira H., Santos S.B., Costa S., Kuyumcu S., Melo L.D.R., Azeredo J. (2019). Synergistic action of phage and antibiotics: Parameters to enhance the killing efficacy against mono and dual-species biofilms. Antibiotics.

[17] Lopatina A., Tal N., Sorek R. (2020). Abortive infection: Bacterial suicide as an antiviral immune strategy. Annu. Rev. Virol..

[18] Rostøl J.T., Marraffini L. (2019). (Ph)ighting phages: How bacteria resist their parasites. Cell Host Microbe.

[19] Chan B.K., Sistrom M., Wertz J.E., Kortright K.E., Narayan D., Turner P.E. (2016). Phage selection restores antibiotic sensitivity in MDR *Pseudomonas aeruginosa*. Sci. Rep..

[20] Engeman E., Freyberger H.R., Corey B.W., Ward A.M., He Y., Nikolich M.P., Filippov A.A., Tyner S.D., Jacobs A.C. (2021). Synergistic killing and re-sensitization of *Pseudomonas aeruginosa* to antibiotics by phage-antibiotic combination treatment. Pharmaceuticals.

[21] Petsong K., Uddin M.J., Vongkamjan K., Ahn J. (2018). Combined effect of bacteriophage and antibiotic on the inhibition of the development of antibiotic resistance in *Salmonella typhimurium*. Food Sci. Biotechnol..

[22] Torres-Barcelo C., Hochberg M.E. (2016). Evolutionary rationale for phages as complements of antibiotics. Trends Microbiol..

[23] Comeau A.M., Tetart F., Trojet S.N., Prere M.F., Krisch H.M. (2007). Phage-Antibiotic Synergy (PAS): Beta-lactam and quinolone antibiotics stimulate virulent phage growth. PLoS ONE.

[24] Lin Y., Chang R.Y.K., Britton W.J., Morales S., Kutter E., Chan H.K. (2018). Synergy of nebulized phage PEV20 and ciprofloxacin combination against *Pseudomonas aeruginosa*. Int. J. Pharm..

[25] Lin Y., Quan D., Chang R.Y.K., Chow M.Y.T., Wang Y., Li M., Morales S., Britton W.J., Kutter E., Li J. (2021). Synergistic activity of phage PEV20-ciprofloxacin combination powder formulation-A proof-of-principle study in a *P. aeruginosa* lung infection model. Eur. J. Pharm. Biopharm..

[26] Davis C.M., Mccutcheon J.G., Dennis J.J. (2021). Aztreonam lysine increases the activity of phages E79 and phiKZ against *Pseudomonas aeruginosa* PA01. Microorganisms.

[27] Kim M., Jo Y., Hwang Y.J., Hong H.W., Hong S.S., Park K., Myung H. (2018). Phage-Antibiotic Synergy via delayed lysis. Appl. Environ. Microbiol..

[28] Kirby A.E. (2012). Synergistic action of gentamicin and bacteriophage in a continuous culture population of *Staphylococcus aureus*. PLoS ONE.

[29] Kaur S., Harjai K., Chhibber S. (2012). Methicillin-resistant *Staphylococcus aureus* phage plaque size enhancement using sublethal concentrations of antibiotics. Appl. Environ. Microbiol..

[30] Kamal F., Dennis J.J. (2015). *Burkholderia cepacia* complex Phage-Antibiotic Synergy (PAS): Antibiotics stimulate lytic phage activity. Appl. Environ. Microbiol..

[31] Abedon S.T., Yin J., Clokie M.R.J., Kropinski A.M. (2009). Bacteriophage plaques: Theory and analysis. Bacteriophages: Methods and Protocols.

[32] Koch A.L. (1964). The growth of viral plaques during the enlargement phase. J. Theor. Biol..

[33] Yin J., Mccaskill J.S. (1992). Replication of viruses in a growing plaque: A reaction-diffusion model. Biophys. J..

[34] Gallet R., Kannoly S., Wang I.-N. (2011). Effects of bacteriophage traits on plaque formation. BMC Microbiol..

[35] Fort J., Méndez V. (2002). Time-delayed spread of viruses in growing plaques. Phys. Rev. Lett..

[36] Ortega-Cejas V., Fort J., Mendez V., Campos D. (2004). Approximate solution to the speed of spreading viruses. Phys. Rev. E.

[37] Abedon S.T., Culler R.R. (2007). Bacteriophage evolution given spatial constraint. J. Theor. Biol..

[38] Hagens S., Habel A., Bläsi U. (2006). Augmentation of the antimicrobial efficacy of antibiotics by filamented phages. Microb. Drug Resist..

[39] Kong K.-F., Schneper L., Mathee K. (2010). Beta-lactam antibiotics: From antibiosis to resistance and bacteriology. APMIS.

[40] Tanaka M., Otsuki M., Nishino T. (1992). *In vitro* and *in vivo* activities of DQ-2556 and its mode of action. Antimicrob. Agents Chemother..

[41] Curtis N.A., Orr D., Ross G.W., Boulton M.G. (1979). Affinities of penicillins and cephalosporins for the penicillin-binding proteins of *Escherichia coli* K-12 and their antibacterial activity. Antimicrob. Agents Chemother..

[42] Horii T., Kobayashi M., Sato K., Ichiyama S., Ohta M. (1998). An *in-vitro* study of carbapenem-induced morphological changes and endotoxin release in clinical isolates of gram-negative *bacilli*. J. Antimicrob. Chemother..

[43] Cushnie T.P., O’driscoll N.H., Lamb A.J. (2016). Morphological and ultrastructural changes in bacterial cells as an indicator of antibacterial mechanism of action. Cell. Mol. Life Sci..

[44] Elliott T.S.J., Shelton A., Greenwood D. (1987). The response of *Escherichia coli* to ciprofloxacin and norfloxacin. J. Med. Microbiol..

[45] Suzuki H., Pangborn J., Kilgore W.W. (1967). Filamentous cells of *Escherichia coli* formed in the presence of mitomycin. J. Bacteriol..

[46] Lewin C., Amyes S. (1991). The role of the SOS response in bacteria exposed to zidovudine or trimethoprim. J. Med. Microbiol..

[47] Nanda A.M., Heyer A., Kramer C., Grunberger A., Kohlheyer D., Frunzke J. (2014). Analysis of SOS-induced spontaneous prophage induction in *Corynebacterium glutamicum* at the single-cell level. J. Bacteriol..

[48] Waldor M.K., Friedman D.I. (2005). Phage regulatory circuits and virulence gene expression. Curr. Opin. Microbiol..

[49] Bergersen F.J. (1953). Cytological changes induced in *Bacterium coli* by chloramphenicol. Microbiology.

[50] Gilleland L.B., Gilleland H.E., Gibson J.A., Champlin F.R. (1989). Adaptive resistance to aminoglycoside antibiotics in *Pseudomonas aeruginosa*. J. Med. Microbiol..

[51] Someya A., Tanaka K., Tanaka N. (1979). Morphological changes of *Escherichia coli* induced by bicyclomycin. Antimicrob. Agents Chemother..

[52] Hadas H., Einav M., Fishov I., Zaritsky A. (1997). Bacteriophage T4 development depends on the physiology of its host *Escherichia coli*. Microbiology.

[53] Rakhuba D., Kolomiets E., Dey E.S., Novik G. (2010). Bacteriophage receptors, mechanisms of phage adsorption and penetration into host cell. Pol. J. Microbiol..

[54] Cahill J., Young R. (2019). Phage lysis: Multiple genes for multiple barriers. Adv. Virus Res..

[55] Kutter E., Sulakvelidze A. (2004). Bacteriophages: Biology and Applications.

[56] Ryan E.M., Alkawareek M.Y., Donnelly R.F., Gilmore B.F. (2012). Synergistic phage-antibiotic combinations for the control of *Escherichia coli* biofilms in vitro. FEMS Immunol. Med. Microbiol..

[57] Easwaran M., De Zoysa M., Shin H.J. (2020). Application of phage therapy: Synergistic effect of phage EcSw (PhiEcSw) and antibiotic combination towards antibiotic-resistant *Escherichia coli*. Transbound. Emerg. Dis..

[58] Abedon S.T., Herschler T.D., Stopar D. (2001). Bacteriophage latent-period evolution as a response to resource availability. Appl Environ. Microbiol..

[59] Bode W. (1967). Lysis inhibition in *Escherichia coli* infected with bacteriophage T4. J. Virol..

[60] Abedon S.T. (1992). Lysis of lysis-inhibited bacteriophage T4-infected cells. J. Bacteriol..

[61] Al-Anany A.M., Fatima R., Hynes A.P. (2021). Temperate phage-antibiotic synergy eradicates bacteria through depletion of lysogens. Cell Rep..

[62] Santos S.B., Carvalho C.M., Sillankorva S., Nicolau A., Ferreira E.C., Azeredo J. (2009). The use of antibiotics to improve phage detection and enumeration by the double-layer agar technique. BMC Microbiol..

[63] Reygaert W.C. (2018). An overview of the antimicrobial resistance mechanisms of bacteria. AIMS Microbiol..

[64] Munita J.M., Arias C.A. (2016). Mechanisms of antibiotic resistance. Microbiol. Spectr..

[65] Hogan D., Kolter R. (2002). Why are bacteria refractory to antimicrobials?. Curr. Opin. Microbiol..

[66] Chow M.Y.T., Chang R.Y.K., Li M., Wang Y., Lin Y., Morales S., Mclachlan A.J., Kutter E., Li J., Chan H.K. (2020). Pharmacokinetics and time-kill study of inhaled antipseudomonal bacteriophage therapy in mice. Antimicrob. Agents Chemother..

[67] Chang R.Y.K., Chow M.Y.T., Wang Y., Liu C., Hong Q., Morales S., Mclachlan A.J., Kutter E., Li J., Chan H.K. (2022). The effects of different doses of inhaled bacteriophage therapy for Pseudomonas aeruginosa pulmonary infections in mice. Clin. Microbiol. Infect..

[68] Chang R.Y.K., Das T., Manos J., Kutter E., Morales S., Chan H.K. (2019). Bacteriophage PEV20 and ciprofloxacin combination treatment enhances removal of *Pseudomonas aeruginosa* biofilm isolated from cystic fibrosis and wound patients. AAPS J..

[69] Sturino J.M., Klaenhammer T.R. (2007). Inhibition of bacteriophage replication in *Streptococcus thermophilus* by subunit poisoning of primase. Microbiology.

[70] Lopes A., Pereira C., Almeida A. (2018). Sequential combined effect of phages and antibiotics on the inactivation of *Escherichia coli*. Microorganisms.

[71] Blasco L., Ambroa A., Lopez M., Fernandez-Garcia L., Bleriot I., Trastoy R., Ramos-Vivas J., Coenye T., Fernandez-Cuenca F., Vila J. (2019). Combined use of the Ab105-2phiDeltaCI lytic putant phage and different antibiotics in clinical isolates of multi-resistant *Acinetobacter baumannii*. Microorganisms.

[72] Chaudhry W.N., Concepcion-Acevedo J., Park T., Andleeb S., Bull J.J., Levin B.R. (2017). Synergy and order effects of antibiotics and phages in killing *Pseudomonas aeruginosa* biofilms. PLoS ONE.

[73] Torres-Barcelo C., Arias-Sanchez F.I., Vasse M., Ramsayer J., Kaltz O., Hochberg M.E. (2014). A window of opportunity to control the bacterial pathogen *Pseudomonas aeruginosa* combining antibiotics and phages. PLoS ONE.

[74] Zuo P., Yu P., Alvarez P.J.J. (2021). Aminoglycosides antagonize bacteriophage proliferation, attenuating phage suppression of bacterial growth, biofilm formation, and antibiotic resistance. Appl. Environ. Microbiol..

[75] Okuyama A., Tanaka N. (1972). Differential effects of aminoglycosides on cistron-specific initiation of protein synthesis. Biochem. Biophys. Res. Commun..

[76] Jiang Z., Wei J., Liang Y., Peng N., Li Y. (2020). Aminoglycoside antibiotics inhibit mycobacteriophage infection. Antibiotics.

[77] Constantinou A., Voelkel-Meiman K., Sternglanz R., Mccorquodale M.M., Mccorquodale D.J. (1986). Involvement of host DNA gyrase in growth of bacteriophage T5. J. Virol..

[78] Jeon G., Ahn J. (2020). Assessment of phage-mediated inhibition of *Salmonella Typhimurium* treated with sublethal concentrations of ceftriaxone and ciprofloxacin. FEMS Microbiol. Lett..

[79] Kumaran D., Taha M., Yi Q., Ramirez-Arcos S., Diallo J.S., Carli A., Abdelbary H. (2018). Does treatment order matter? Investigating the ability of bacteriophage to augment antibiotic activity against *Staphylococcus aureus* biofilms. Front. Microbiol..

[80] Wang L., Tkhilaishvili T., Trampuz A. (2020). Adjunctive use of phage Sb-1 in antibiotics enhances inhibitory biofilm growth activity versus rifampin-resistant *Staphylococcus aureus* strains. Antibiotics.

[81] Goldstein B.P. (2014). Resistance to rifampicin: A review. J. Antibiot..

[82] Wang L., Tkhilaishvili T., Bernal Andres B., Trampuz A., Gonzalez Moreno M. (2020). Bacteriophage-antibiotic combinations against ciprofloxacin/ceftriaxone-resistant *Escherichia coli* in vitro and in an experimental *Galleria mellonella* model. Int. J. Antimicrob. Agents.

[83] Liu C.G., Green S.I., Min L., Clark J.R., Salazar K.C., Terwilliger A.L., Kaplan H.B., Trautner B.W., Ramig R.F., Maresso A.W. (2020). Phage-Antibiotic Synergy is driven by a unique combination of antibacterial mechanism of action and stoichiometry. MBio.

[84] Thornton L.A., Burchell R.K., Burton S.E., Lopez-Villalobos N., Pereira D., Macewan I., Fang C., Hatmodjo A.C., Nelson M.A., Grinberg A. (2018). The effect of urine concentration and pH on the growth of *Escherichia coli* in canine urine in vitro. J. Vet.-Intern. Med..

[85] Pacios O., Fernández-García L., Bleriot I., Blasco L., González-Bardanca M., López M., Fernández-Cuenca F., Oteo J., Pascual Á., Martínez-Martínez L. (2021). Enhanced antibacterial activity of repurposed mitomycin C and imipenem in combination with the lytic phage vB_KpnM-VAC13 against clinical isolates of *Klebsiella pneumoniae*. Antimicrob. Agents Chemother..

[86] Oechslin F., Piccardi P., Mancini S., Gabard J., Moreillon P., Entenza J.M., Resch G., Que Y.A. (2017). Synergistic interaction between phage therapy and antibiotics clears *Pseudomonas Aeruginosa* infection in endocarditis and reduces virulence. J. Infect. Dis..

[87] Kaur S., Harjai K., Chhibber S. (2016). In vivo assessment of phage and linezolid based implant coatings for treatment of methicillin resistant *S. aureus* (MRSA) mediated orthopaedic device related infections. PLoS ONE.

[88] Chhibber S., Kaur T., Sandeep K. (2013). Co-therapy using lytic bacteriophage and linezolid: Effective treatment in eliminating methicillin resistant *Staphylococcus aureus* (MRSA) from diabetic foot infections. PLoS ONE.

[89] Tan X., Chen H., Zhang M., Zhao Y., Jiang Y., Liu X., Huang W., Ma Y. (2021). Clinical experience of personalized phage therapy against carbapenem-resistant *Acinetobacter baumannii* lung infection in a patient with chronic obstructive pulmonary disease. Front. Cell. Infect. Microbiol..

[90] Roach D.R., Leung C.Y., Henry M., Morello E., Singh D., Di Santo J.P., Weitz J.S., Debarbieux L. (2017). Synergy between the host immune system and bacteriophage is essential for successful phage therapy against an acute respiratory pathogen. Cell Host Microbe.

[91] Gainey A.B., Burch A.-K., Brownstein M.J., Brown D.E., Fackler J., Horne B.A., Biswas B., Bivens B.N., Malagon F., Daniels R. (2020). Combining bacteriophages with cefiderocol and meropenem/vaborbactam to treat a pan-drug resistant *Achromobacter* species infection in a pediatric cystic fibrosis patient. Pediatr. Pulmonol..

[92] Moulton-Brown C.E., Friman V.P. (2018). Rapid evolution of generalized resistance mechanisms can constrain the efficacy of phage-antibiotic treatments. Evol. Appl..

[93] Buckling A., Rainey P.B. (2002). Antagonistic coevolution between a bacterium and a bacteriophage. Proc. Biol. Sci..

[94] Cairns J., Becks L., Jalasvuori M., Hiltunen T. (2017). Sublethal streptomycin concentrations and lytic bacteriophage together promote resistance evolution. Philos. Trans. R. Soc. Lond. B Biol. Sci..

[95] Barnard A.M.L., Fairhead H.I.M. (2021). A commentary on the development of engineered phage as therapeutics. Drug Discov. Today.

[96] Kebriaei R., Lev K.L., Stamper K.C., Lehman S.M., Morales S., Rybak M.J. (2020). Bacteriophage AB-SA01 cocktail in combination with antibiotics against MRSA-VISA strain in an in vitro pharmacokinetic/pharmacodynamic model. Antimicrob. Agents Chemother..

[97] Luscher A., Simonin J., Falconnet L., Valot B., Hocquet D., Chanson M., Resch G., Kohler T., Van Delden C. (2020). Combined bacteriophage and antibiotic treatment prevents *Pseudomonas aeruginosa* infection of wild type and cftr-epithelial cells. Front. Microbiol..

[98] Kaur P., Gondil V.S., Chhibber S. (2019). A novel wound dressing consisting of PVA-SA hybrid hydrogel membrane for topical delivery of bacteriophages and antibiotics. Int. J. Pharm..

[99] Lin Y., Chang R.Y.K., Britton W.J., Morales S., Kutter E., Li J., Chan H.K. (2019). Inhalable combination powder formulations of phage and ciprofloxacin for *P. aeruginosa* respiratory infections. Eur. J. Pharm. Biopharm..

[100] Gorski A., Borysowski J., Miedzybrodzki R. (2020). Phage therapy: Towards a successful clinical trial. Antibiotics.

[101] Mutti M., Corsini L. (2019). Robust approaches for the production of active ingredient and drug product for human phage therapy. Front. Microbiol..

